# CD147 silencing inhibits tumor growth by suppressing glucose transport in melanoma

**DOI:** 10.18632/oncotarget.11415

**Published:** 2016-08-19

**Authors:** Juan Su, Tianyuan Gao, Minghao Jiang, Lisha Wu, Weiqi Zeng, Shuang Zhao, Cong Peng, Xiang Chen

**Affiliations:** ^1^ Department of Dermatology, Xiangya Hospital, Central South University, Changsha, Hunan, China; ^2^ Hunan Key Laboratory of Skin Cancer and Psoriasis, Xiangya Hospital, Central South University, Changsha, Hunan, China; ^3^ Department of Dermatology, the First Affiliated Hospital of Xi’an Jiaotong University, Xi’an, Shanxi; ^4^ Department of Anesthesiology, Xiangya Hospital, Central South University, Changsha, Hunan, China; ^5^ Institute of Medical Science Research, Xiangya Hospital, Central South University, Hunan, China

**Keywords:** melanoma, CD147, glycolysis, GLUT-1, PI3K/Akt pathway

## Abstract

Melanoma is a very malignant disease and there are still no effective treatments. CD147 participates in the carcinogenesis of multiple human cancers and GLUT-1, as a glucose transporter, is associated with tumor growth. However, the function of CD147 and GLUT-1 in melanoma have not been completely understood. Thus, in this study we investigated the expression of CD147 and GLUT-1 in melanoma tissue, which were overexpressed compared with that in nevus tissue. In addition, CD147 and GLUT-1 were co-localized in the cytoplasm of human melanoma A375 cells. Immunoprecipitation proved that CD147 interacted with GLUT-1 at D105-199. Silencing CD147 by specific siRNA could downregulate GLUT-1 level via inhibiting PI3K/Akt signaling and decrease glucose uptake in A375 cells. *In vivo* experiments also supported that CD147 knockdown suppressed the tumor growth in melanoma subcutaneous mice model, observed by micro PET/CT. Our results could help validate CD147 as a new therapeutic target for treating melanoma.

## INTRODUCTION

Melanoma is one of the highly malignant and progressive tumors, and the incidence and mortality have been on the increase year by year [1]. In the USA, melanoma was the sixth commonest malignant tumor [2]. Most patients with this deadly disease are associated with poor prognosis and the median survival was around 6 months. Light-colored skin or hair, photosensitivity, sunlight exposure and positive family history could increase the risk for melanoma [3]. However, the potential molecular mechanism for melanoma has not been completely understood, and there are still no effective treatments available.

Glycolysis could provide energy for the tumor growth. Recent studies have proved that the regulation of glycolysis might exert anti-tumor activities in multiple human cancers [4]. Glucose transporter (GLUT) family is kind of glucose transporter protein, which was overexpressed in tumor tissue [5]. GLUT-1 as one member of the GLUT family is a transmembrane glycoprotein located on cell membrane and mediates the transportation of glucose. It was reported that GLUT-1 was upregulated in melanoma and was correlated with the tumor differentiation [6]. In addition, the expression of GLUT-1 may predict the hypoxia and glycolysis in tumor tissue as well as the patients’ outcome. Recently, CD147 has been shown to participate the development and progression of melanoma [7] and is associated with the malignant phenotype, but whether CD147 could regulate the glucose transport in melanoma is still unknown. Thus, in this study we investigated the effect of CD147 silencing on the glucose transport and its interaction with GLUT in melanoma. Our results may help expand the current understanding on the carcinogenesis of melanoma.

## RESULTS

### CD147 was correlated with GLUT-1 in melanoma

**Figure 1:**
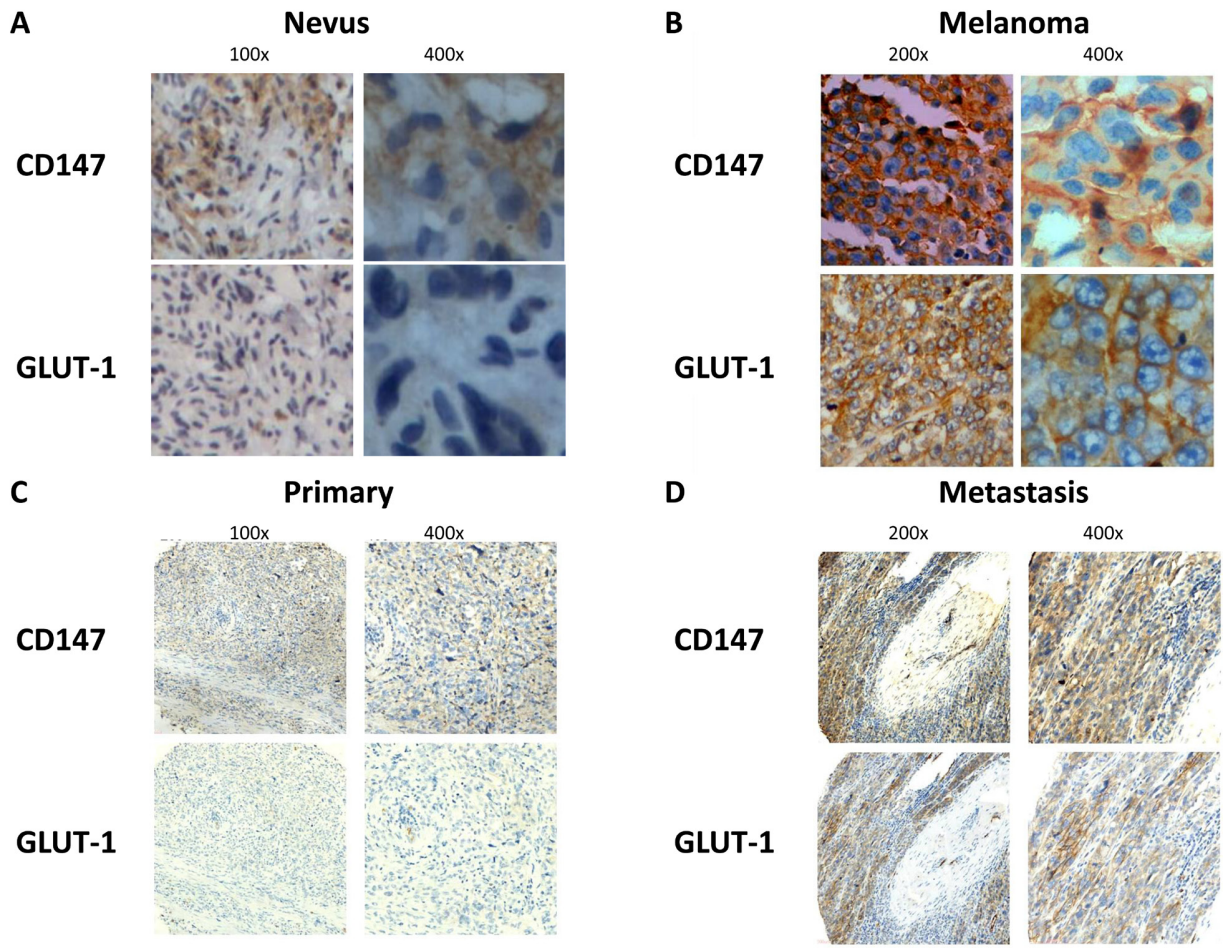
CD147 and GLUT-1 were overexpressed in melanoma compared with nevus **A.**-**B.**, which was upregulated in metastatic melanoma tissue compared with primary melanoma tissue **C.-D.**

We first investigated the expression of CD147 and GLUT-1 in nevus (*n* = 20) and melanoma (*n* = 20) tissue. IHC analysis showed that both CD147 and GLUT-1 were overexpressed in melanoma tissue compared with nevus tissue (*P* = 0.003 for CD147, *P* = 0.002 for GLUT-1; Table 1, Figure 1A-1B). Our results also supported that metastatic melanoma was associated with higher CD147 and GLUT-1 level than primary melanoma (*P* = 0.006 for CD147, *P* = 0.036 for GLUT-1; Table 2, Figure 1C-1D), indicating that CD147 and GLUT-1 might play an important role in the development and progression of melanoma.

**Table 1:** CD147 and GLUT-1 were overexpressed in melanoma

	Nevus (*n*= 22)		Melanoma (*n*= 22)		X^2^	*P* value
	Low	High	Low	High		
CD147	12	10	2	20	10.476	0.003
GLUT-1	15	7	4	18	11.208	0.002

### CD147 interacted with GLUT-1 in A375 cells

**Figure 2:**
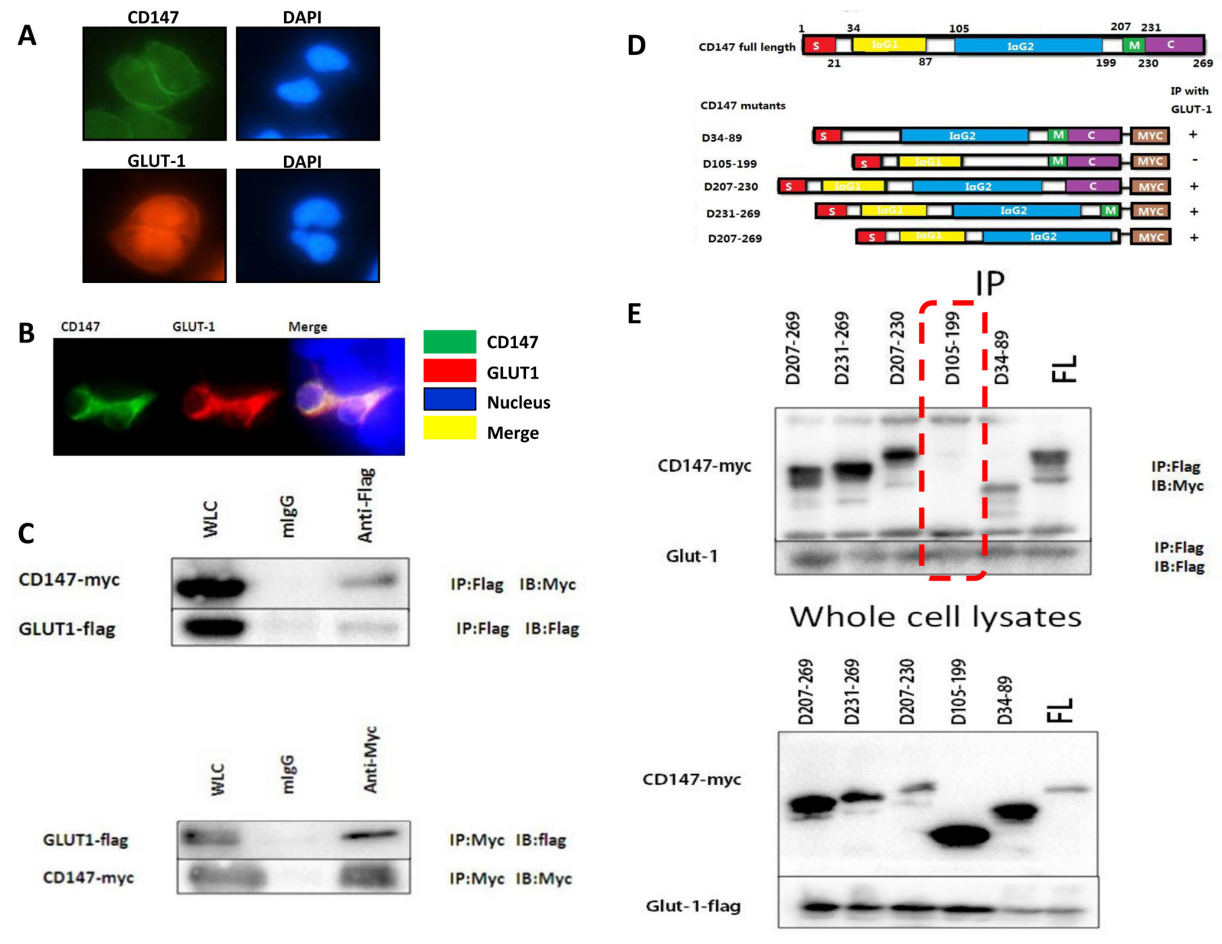
The co-expression of CD147 and GLUT-1 was observed in A375 cells **A.-B.** Co-immunoprecipitation proved that CD147 could interact with GLUT-1 in melanoma at D105-199 **C.**-**E.**.

In order to examine the function of CD147 and GLUT-1, we then detected the localization of these two molecules in human melanoma A375 cells (Figure 2A-2B). Immunofluorescence demonstrated that CD147 and GLUT-1 was co-expressed in the cytoplasm of A375 cells *in vitro* culture. Plasmids expressing CD147-Myc and GLUT-1-Flag were constructed and transfected into 293FT cells. Immunoprecipitation proved that CD147 interacted with GLUT-1 (Figure 2C). Then a total of 5 CD147 deletion variants were synthesized and D34-89, D105-199, D207-230, D231-269 and D207-269 were knockdown (Figure 2D). Immunoprecipitation showed that D105-199 silencing blocked the interaction of CD147 with GLUT-1 (Figure 2E).

**Table 2:** High CD147 and GLUT-1 level was observed in metastatic melanoma compared with primary melanoma

	Primary melanoma (*n*= 127)		Metastatic melanoma (*n*= 62)		X^2^	*P* value
	Low	High	Low	High		
CD147	45	82	10	52	7.525	0.006
GLUT-1	48	79	14	48	4.375	0.036

### CD147 silencing downregulated GLUT-1 *via* PI3K/AKT pathway

**Figure 3:**
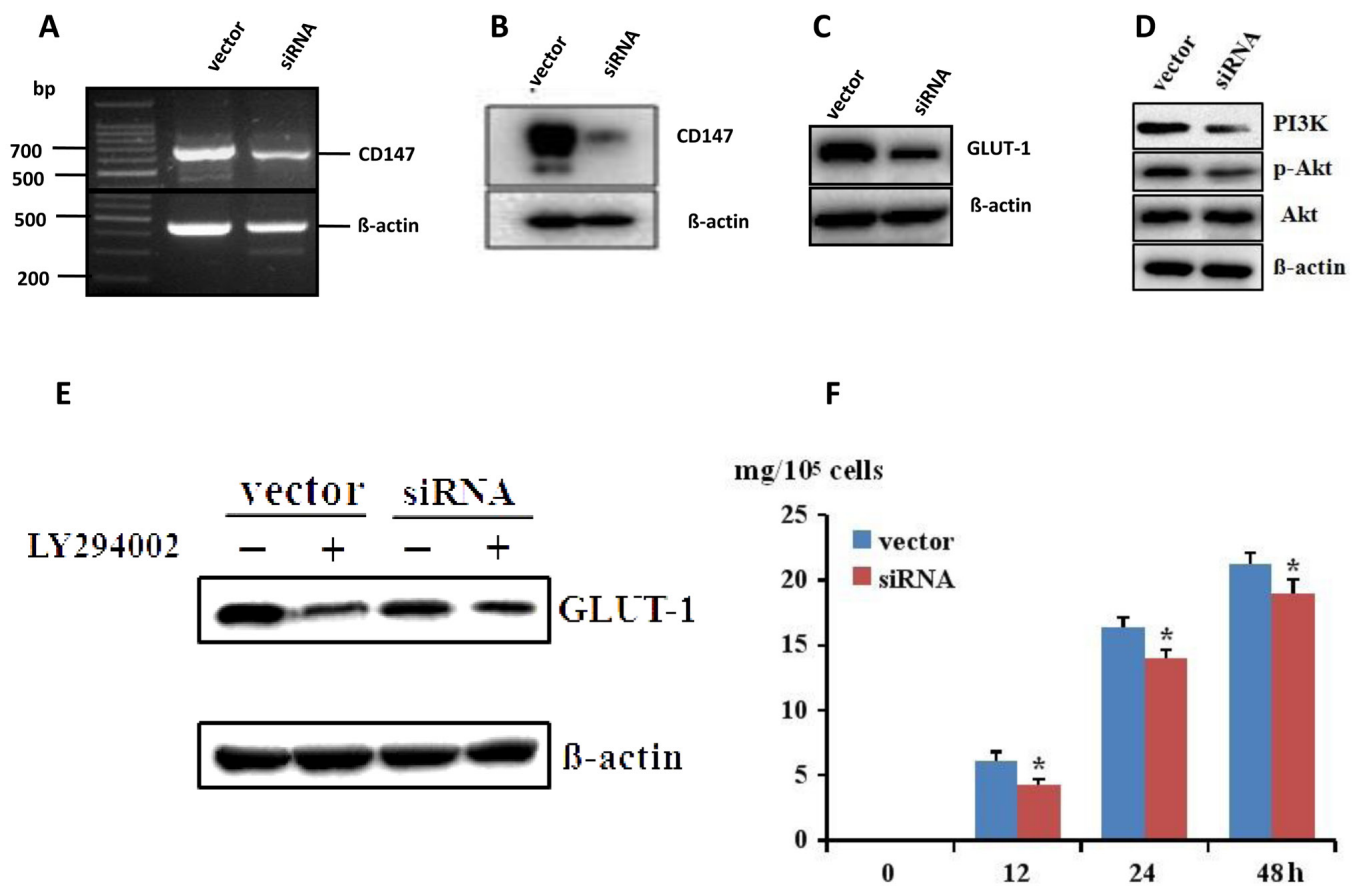
CD147 mRNA and protein level was decreased after transfection with CD147 siRNA in A375 cells **A.**-**B.**, which lead to the downregulation of GLUT-1 *via* PI3K/Akt pathway **C.**-**D.** PI3K inhibitor LY294002 could abolish the inhibition of CD147 silencing on GLUT-1 **E.** The glucose uptake was downregulated after CD147 knockdown **F.** *p < 0.05 compared with the vector.

As IHC proved that CD147 and GLUT-1 were both overexpressed in melanoma tissue, we hypothesized that CD147 might regulate the expression of GLUT-1. It was observed that GLUT-1 level was decreased in A375-shCD147 cells compared with that in A375 cells (Figure 3A-3C). The PI3K and p-Akt level were also downregulated by CD147 silencing (Figure 3D). LY294002, which is a specific inhibitor for PI3K/Akt signaling, could abolish the inhibitory effect of CD147 silencing on the GLUT-1 level (Figure 3E). The glucose uptake was also decreased in A375-shCD147 cells (Figure 3F), suggesting that CD147 silencing may impair the glucose supply, which is required for tumor growth in melanoma.

### Targeting CD147 could suppress tumor growth and lung metastasis in melanoma

**Figure 4:**
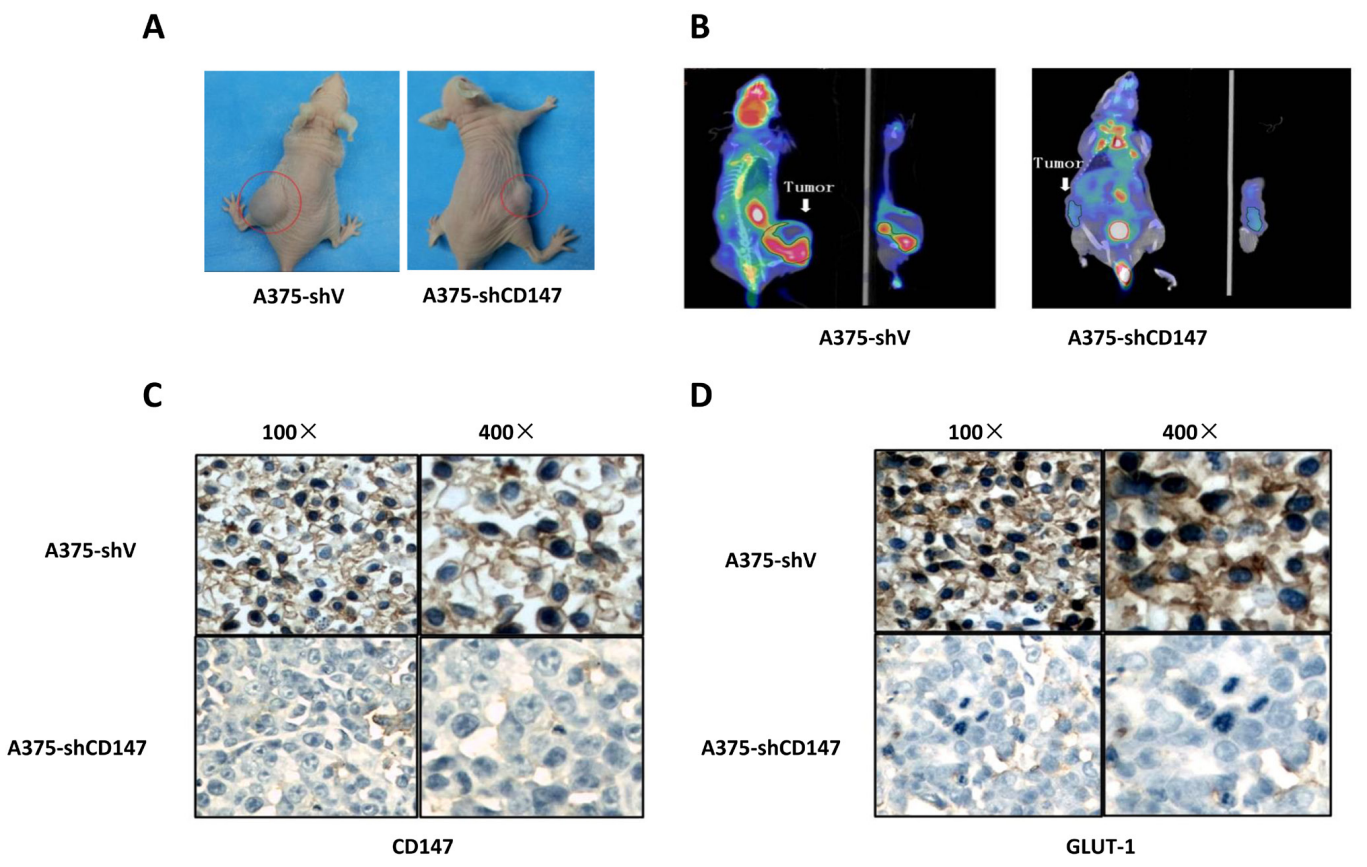
Decreased F-FDG uptake was observed by micro PET/CT in CD147 knockdown mice model **A.**, and CD147 knockdown downregulated GLUY-1 level in mice model **B.**

The inhibition of CD147 silencing on melanoma was further investigated in subcutaneous mice model. Micro PET/CT scan observed that mean SUV value was 1.53±0.11 in A375 group (*n* = 10) and 0.45±0.07 in A375-shCD147 group (*n* = 10), respectively (*P* < 0.05; Figure 4A-4B). IHC analysis of the tumor tissue showed that GLUT-1 level was downregulated (Figure 4C-4D). In melanoma metastasis mice model, less lung metastatic lesions were found in A375-shCD147 group comparing with those in A375 group (*P* < 0.05).

## DISCUSSION

Our study for the first time reported that CD147 silencing could suppress the tumor growth by downregulating the level of GLUT-1 in melanoma. CD147 and GLUT-1 were both overexpressed in melanoma tissue and positively correlated. CD147 could modulate the expression of GLUT-1 *via* PI3K/Akt pathway and also interact with GLUT-1, which is an important glucose transporter.

Melanoma is highly malignant with very poor prognosis [8]. Although great effort has been made on understanding the carcinogenesis of melanoma, there are still not effective treatment [9, 10]. Vemurafenib (Zelboraf, Roche) and dabrafenib (Tafinlar, GlaxoSmithKline) are two BRAF inhibitors, which has been applied in the treatment for melanoma [11, 12], but the survival rate remains low. Thus, it is important to identify new therapeutic targets in order to improve the clinical outcome for such patients with this deadly disease. In 1930, Warburg first found that glycolysis was common in tumor cells even under normoxia, which may be explained by the fact that tumor cells could uptake plenty of glucose and glycolysis can provide sufficient energy for maintaining the tumor growth [13]. This metabolic activity of tumor cells has attracted great attentions from researchers all over the world. Recent studies have reported that lactate dehydrogenase (LDH), pyruvate dehydrogenase (PDK) and hexokinase (HK) could serve as new therapeutic targets for treating human cancers [14]. GLUT family members facilitate the transportation of glucose into cells and GLUT-1 level was proved to be associated with oncogenic gene, growth factors, interleukin-1, local hypoxia and matrix metalloproteinases [15-17]. GLUT-1 overexpression was observed in multiple human cancers, which was correlated with the invasive phenotype [18-20]. Thus, impairing the function of GLUT-1 might be introduced as a new anti-tumor therapy.

In this study, we investigated the interaction of CD147 and GLUT-1. CD147 silencing could also downregulate the level of GLUT-1 and decreased glucose uptake was observed in A375-shCD147 cells. *In vivo* experiments also supported that CD147 silencing could inhibit the tumor growth and lung metastasis in subcutaneous mice model. PI3K/Akt signaling was involved in the regulation of CD147 on GLUT-1 in melanoma cells. Previous studies has already verified that CD147 could participate in a series of biological processes including cell proliferation, invasion and migration by inducing the production of matrix metalloproteinases [21-24]. So targeting CD147 is able to suppress the tumor growth as well as to impair the interaction between tumor cells and stroma cells. It was also proved that PI3K/Akt signaling was increased in the transformation from benign nevus to malignant melanoma [9, 25].

Taken together, our results demonstrated that CD147 knockdown could suppress the tumor growth of melanoma by downregulating GLUT-1 *via* PI3K/Akt pathway, which could validate CD147 silencing by specific siRNAs as a new therapeutic regimen for treating melanoma. However, the anti-tumor action and efficacy of CD147 silencing in melanoma should be further elaborated.

## MATERIALS AND METHODS

### Cell culture and tissue

Human melanoma A375 cells and 293FT cells were purchased from American Type Culture Collection (ATCC). Cells were cultured in DMEM with 1% penicillin, 1% streptomycin and 10% fetal bovine serum at 5% CO_2_ and 37°C. Tissues of melanoma and pigmented nevus were collected from age- and gender- matched patients who underwent resection in our department. Our study was approved by the Ethic Committee of Zhongnan University in accordance with Declaration of Helsinki and informed consent was obtained from all the patients.

### Transfection and real time RT-PCR

Specific siRNA targeting CD147 was designed and synthesized. A375 cells were transfected with CD147 siRNA and scramble siRNA (control) by lipofectamine 2000, respectively. After transfection, cells were collected and RNA was extracted. Stable A375-shV and A375-shCD147 cell lines were established by being transfected with constructed plasmid harboring scramble and CD147 shRNA, respectively. Real time RT-PCR was conducted to detect the mRNA level of CD147 and β-actin was used to be normalized as internal control.

### Western blot

Protein sample was prepared from total cell lysate and quantified using BCA kit. 10ul protein sample was loaded in the SDS-PAGE gel for electrophoresis, which was then transferred to PVDF membrane. The blot was blocked in milk and primary antibody for detecting CD147, PI3K, p-Akt, Akt, GLUT-1 and GLUT4 were used at the dilution ratio of 1:500. β-actin served as internal control.

### Immunoprecipitation and immunofluorescence

Protein A+G agarose beads was used in the immunoprecipation to detect the interaction of CD147 and GLUT-1 and CD147 deletion variants were constructed to determine the binding site with GLUT-1. Different CD147 deletion variants with Myc tag were introduced into 293FT cells. Protein sample, cells and tissues were all first incubated in primary antibody 1:200 anti-CD147 and then secondary antibody including Cy3 1:300, FITC 1:500 and Cy5 1:300, which was observed by fluorescence microscope.

### Melanoma nude mice model and micro PET/CT

A375-shV and A375-shCD147 cells were injected subcutaneously into 4-6 weeks female nude mice at the density of 10^7^/0.2ml. The tumor volume was measured and recorded. After the establishment of the mice model, 0.2mL ^18^F-FDG was injected by tail vein. Then the mice was scanned by Siemens Inveon micro PET/CT. The imaging was analyzed by Inveon Acquisition Workplace (IAW) and the region of interest (RIO) was obtained. Standard uptake value (SUV) of RIO was tested by one-way ANOVA. The melanoma metastasis mice model was also established by injecting tumor cells *via* tail vein. After 6 weeks, the mice were sacrificed and lung tissue was collected.

### Immunohistochemical analysis

Tumor tissue was fixed in paraffin and cut into 5-μm slices. 1:500 Anti-CD147 and anti-GLUT-1 antibodies were used as primary antibodies. Two pathologist evaluated all the slices independently. A total of 5 images under high power field were randomly selected for each slice. The staining could categorized into 0 (no stain), 1 (light brown), 2 (brown) and 3 (dark brown) and the positivity was classified into 0 (0%), 1 (1-25%), 2 (26-50%) and 3 ( > 50%). The averaged score (0-9) was based on the staining score x the positivity score, and classified into 1 (0), 1 ( < 3), 2 (3-6) and 3 ( > 6). 0 and 1 were considered as low expression and 2 and 3 were as high expression.

### Statistical analysis

SPSS Statistics 17.0 software (SPSS Inc, Chicago, IL) was used for all the analysis. For categorical data, Pearson chi-square test was applied for detecting the difference. Difference on continuous data was tested by independent student *t*-test. The correlation of CD147 expression and GLUT-1 level was tested by Spearman correlation analysis. P value less than 0.05 was considered to be statistically significant.
